# Meeting Upcoming Clinical and Diagnostic Needs in Oncologic Imaging: A Structured Reporting System for Fibroblast-Activation-Protein–Targeted Imaging—FAP-RADS Version 1.0

**DOI:** 10.2967/jnumed.125.269914

**Published:** 2025-08

**Authors:** Emil Novruzov, Gabriel T. Sheikh, Eduards Mamlins, Adrien Holzgreve, Yuriko Mori, Stephan Ledderose, Frederik Klauschen, Tadashi Watabe, Michael A. Gorin, Martin G. Pomper, Ken Herrmann, Steven P. Rowe, Rudolf A. Werner, Frederik L. Giesel

**Affiliations:** 1 Department of Nuclear Medicine, Medical Faculty and University Hospital Duesseldorf, Heinrich-Heine-University Duesseldorf, Düsseldorf, Germany;; 2 Department of Nuclear Medicine, LMU University Hospital, LMU Munich, Munich, Germany;; 3 Institute of Pathology, Ludwig Maximilians University Munich, Germany;; 4 Department of Radiology, Graduate School of Medicine, The University of Osaka, Osaka, Japan;; 5 Milton and Carroll Petrie Department of Urology, Icahn School of Medicine at Mount Sinai, New York, New York;; 6 Department of Radiology, University of Texas Southwestern Medical Center, Dallas, Texas;; 7 Department of Nuclear Medicine, University of Duisburg-Essen, Essen, Germany; German Cancer Consortium, University Hospital Essen, Essen, Germany;; 8 Department of Radiology, University of North Carolina, Chapel Hill, North Carolina;; 9 The Russell H. Morgan Department of Radiology and Radiological Science, Division of Nuclear Medicine and Molecular Imaging, Johns Hopkins School of Medicine, Baltimore, Maryland; and; 10 Center for Integrated Oncology Aachen Bonn Cologne Düsseldorf, Düsseldorf, Germany

**Keywords:** FAP-RADS, FAP PET, FAP-targeted imaging, FAPI, standardized reporting, RADS framework

## Abstract

Fibroblast activation protein (FAP)–targeted imaging has emerged as a promising diagnostic tool for various oncologic and nononcologic conditions. However, the increasing employment of FAP-targeted imaging with small-molecule radiotracers mandates a standardized reporting system to ensure consistent interpretation across various clinical scenarios, especially in oncologic imaging. This study proposes an FAP reporting and data system (RADS) framework (FAP-RADS version 1.0) to address those needs, aiming to standardize FAP-targeted imaging reports, improve clinical communication, and support multicenter research. **Methods:** We conducted a comprehensive literature review and combined the findings with expert consensus in nuclear medicine to design a new scoring system based on the principles of the molecular imaging RADS framework. FAP-RADS version 1.0 is intended for use across multiple tumor types with variable radiotracer uptake patterns and is nonspecific to imaging modality (FAP PET and FAP SPECT) and radiotracer compound structure. We aimed to ensure that the system would be user-friendly and applicable to routine clinical practice. **Results:** FAP-RADS version 1.0 introduces a 5-point scale to assess the likelihood of malignancy in lesions based on the imaging characteristics of the lesions. The scoring system can be applied to different FAP ligands with varying pharmacokinetics. The framework does not rely on SUV thresholds, making it flexible and robust for clinical use. The implementation of this system aims to improve interdisciplinary communication, support longitudinal follow-up, and facilitate future multicenter trials. **Conclusion:** The proposed FAP-RADS version 1.0 is a comprehensive and flexible framework that has the potential to enhance the quality and consistency of FAP-targeted imaging interpretation in clinical practice. Its application may lead to better research outcomes, broader acceptance within the oncologic community, and streamlined regulatory processes related to FAP-based radiotracer approval.

## Visual Abstract

**Figure:**
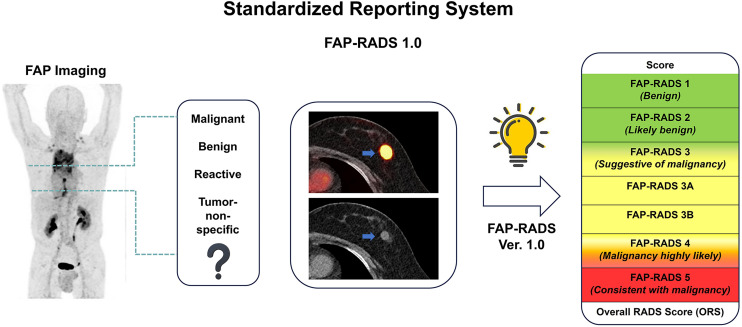

Fibroblast activation protein (FAP) is a transmembrane glycoprotein that is overexpressed on cancer-associated fibroblasts of epithelial malignancies. FAP has been shown to play a pivotal role in drug resistance, immunosurveillance, and tumor invasion and growth by remodeling the tumor microenvironment. FAP is also upregulated in several fibrotic and inflammatory processes, such as degenerative changes in joints or wound healing, and scarcely expressed in normal tissue (1).

After several trials using antibody-mediated or chimeric antigen receptor T-cell–mediated FAP targeting, a pharmacophore appropriate for the development of small-molecule imaging agents was developed and preclinically investigated by a group at Johns Hopkins University School of Medicine (2). The Heidelberg research group developed similar agents for oncologic use (3–5). These compounds were able to selectively bind and inhibit the enzymatic portion of the FAP target and are thus termed *FAP inhibitors* (FAPIs). FAPIs exhibit a high affinity for FAP, enhancing the binding capacity and internalization of the ligand-receptor complex, with implications for both imaging and therapeutic applications.

Although the backbone of FAP imaging encompasses the use of either ^68^Ga- or ^18^F-labeled FAP PET tracers, there have been attempts to establish FAP SPECT agents that leverage ^99m^Tc. The quinoline-based FAPI agents typically exhibit a rapid tumor uptake and likewise demonstrate relatively rapid washout. FAPIs labeled with radionuclides with long half-lives could be applied by using alternative therapy protocols (3–5). Novel FAP-binding radiotracers using cyclic peptides as binding motifs, such as FAP-2286, might serve as an alternative (6,7).

The growing body of evidence for several FAPI derivatives has underscored the potential of FAP as a novel pan-cancer imaging target. Previous studies have shown the superiority of FAP-targeted PET agents over [^18^F]FDG for a variety of entities, such as gastrointestinal tumors and peritoneal carcinomatosis (8,9). FAP-targeted imaging has applications in primary staging, therapeutic monitoring, and recurrence work-up. Given the ongoing pivotal phase 2 and 3 trials (10–12), we anticipate the upcoming inclusion of FAPI derivatives in national and international guidelines for various indications.

The increasing use of FAP-targeted imaging highlights challenges related to the nonspecific uptake of FAP agents, as certain benign processes may also exhibit varying levels of FAPI uptake, potentially leading to pitfalls. There is currently no framework that can be used by molecular imaging experts to stratify nononcologic lesions. Moreover, the vast number of oncologic entities for which FAPI may have applications exhibits heterogeneous uptake patterns, depending on the degree of FAP upregulation in the specific tumor entity (13). Despite the superiority of diagnostic performance according to initial data, the clinical impact of FAP-targeted imaging still requires thorough investigation (14). Those unmet needs mandate the employment of a unified language across research and clinical centers regarding the interpretation and reporting of data for both research and clinical purposes to ensure reproducibility, provide reliability, and ultimately enhance the acceptance of FAP-targeted imaging.

Here, we present a structured system for interpreting FAP-targeted imaging reports, providing a standardized approach for research and clinical practice. This proposal has been developed through a combination of expertise across institutions in the United States and Europe and is informed by a systematic analysis of the existing literature in standardized reporting of molecular imaging studies in nuclear medicine, as well as what is known about the discrimination between malignant and benign findings on FAP-targeted imaging.

## MATERIALS AND METHODS

All procedures performed in studies involving human participants were approved by the ethics committee and performed in accordance with the ethical standards of the institutional or national research committees and with the 1964 Helsinki declaration and its later amendments or comparable ethical standards.

### Data Source and Search Strategy

We performed a comprehensive narrative literature review to facilitate solid evidence acquisition and synthesis and mitigate any arbitrariness of our proposal. A comprehensive search of an English-language, electronic database (PubMed) was performed by 2 independent investigators between January 2018 and February 2025, using the following index terms: *FAPI PET uptake patterns*, *fibroblast activation protein imaging equivocal uptake*, *fibroblast activation protein imaging semiquantitative*, *FAP imaging discrimination*, *FAPI PET distinguishing benign and malignant uptake*, *FAPI PET benign uptake*, and *FAPI PET malignant uptake*. While this approach incorporates elements of systematic evidence acquisition, it remains a narrative synthesis rather than a formal systematic review.

### Data Extraction and Quality Assessment

We included relevant original research articles, systematic reviews, and meta-analyses that provided clinical or imaging-based evidence on FAPI PET uptake patterns, relying on Preferred Reporting Items for Systematic Reviews and Meta-Analyses (better known as PRISMA) (15). Non-English-language articles, preclinical studies, preprint manuscripts, and nonoriginal publications (e.g., case reports, letters, duplicate publications, general reviews) were excluded. We evaluated the preselected works to maintain a definitive process of evidence synthesis.

## RESULTS

### Normal Organ Biodistribution of FAP Ligands

The evidence indicated a high rate of consistency regarding the biodistribution and tumor uptake of various FAPI ligands, unless the uptake time between radiotracer administration and image acquisition exceeded 60 min after injection (16–19). The biodistribution studies showed a low FAPI signal in most normal organs and tissues, such as lung parenchyma, esophagus, left ventricle walls, nipples, glandular breast tissue, pancreas, renal cortex, parathyroid gland, adrenal gland, oral mucosa, stomach, duodenum, small intestine, liver, ovaries, adipose tissue, spleen, tonsils, and bone marrow. Interestingly, some of those organs are considered to lack FAP expression (i.e., parathyroid gland, adrenal gland, oral mucosa, stomach, duodenum, small intestine, liver, pancreas, ovaries, adipose tissue, spleen, tonsils, and bone marrow). FAPI uptake in those organs seemed to arise from the high similarity of dipeptidyl peptidase IV with FAP. As a member of prolyl peptidase family, FAP protein shares 70% similarity of its amino acid sequence with dipeptidyl peptidase IV (20,21).

The only normal organ with moderate-to-high background uptake (SUV_max_ > 9.6) appeared to be the corpus uteri because of high FAP expression in endometrial glandular cells. Moreover, FAPI uptake has been shown to vary with the menstrual cycle (22). Since FAPI uptake in normal uterine tissue was shown to persist into the postmenopausal period (SUV_max_ > 6), accurate detection and delineation of primary lesions were challenging in endometrial lesions. Notably, the detection of primary cervical cancer proved less challenging because of more favorable FAPI uptake in the uterine cervix (SUV_max_ 5.1) (22).

### Stratification of FAPI Uptake Using Semiquantitative Parameters in Malignant Lesions

Several studies have investigated the utility of semiquantitative parameters for accurately discriminating between benign and malignant lesions, as FAP expression is also observed in inflammatory and fibrotic processes, alongside epithelial malignancies (23). The reader must often differentiate FAPI-avid benign lesions in a variety of systems, such as lung, pancreas, colon, and liver, as well as postinterventional changes. In a retrospective study with 44 patients, Shu et al. (24) investigated FAPI radiotracer uptake in benign lesions in various organs and found heterogeneous uptake with a wide range of SUV_max_. The authors reported the necessity for considering the logical features of the CT scan or certain clinical tests to help in the differential diagnosis of such lesions.

Zheng et al. (25) investigated the semiquantitative FAPI uptake pattern in a larger cohort of 146 patients with 186 primary malignant tumors and 360 benign lesions. The benign lesions included inflammatory processes, exostoses, hemorrhoids, fractures, and hepatic fibrosis, with a median SUV_max_ of 3.6 (range, 1.3–21.5), whereas the malignant lesions had a median SUV_max_ of 9.0 (range, 0.9 to 25.7). Even though the median SUV_max_ of benign lesions was low, the authors noted a considerable overlap of SUV_max_ between benign and malignant lesions. A similar conclusion was drawn by Qin et al. (26) after an investigation of malignant and benign bone lesions (mean SUV_max_ of 7.14 ± 4.33 vs. 3.57 ± 1.60, respectively; *P* < 0.001), where the SUV_max_ had notable overlap between lesion types. Wang et al. (27) underscored the importance of the combination of semiquantitative parameters (such as SUV_max_) with the extent of tracer uptake for an accurate stratification for suspected periprosthetic infection, whereas the evaluation of SUV_max_ only could be misleading.

For both [^18^F]FDG and FAPI imaging, postinterventional changes after radiotherapy or surgery present interpretive challenges. Intensity and extent of tracer uptake, as well as time interval after the medical intervention, play roles in correct lesion interpretation. The most frequently encountered challenges include suture granulomas, the inguinal region after cardiovascular interventions, and the abdominal wall after laparotomy, as well as surgical mesh, breast implants, infusion ports, and medical foreign body (FB) implantations. Maliha et al. (28) investigated FAPI uptake in patients with invasive postinterventional changes and implanted medical FBs. The authors observed no FAPI uptake 8 mo after medical interventions without FB implantation, whereas FAPI uptake did not seem to lessen after medical interventions involving FB implantation or after radiotherapy (range, 1–23 y). The presence of FBs was the strongest predictor of nonspecific FAPI uptake.

Liu et al. (29) conducted a study with a cohort of 53 patients involving a total of 262 bone lesions, including the appendicular skeleton and joints. Bone lesions with inflammatory components, such as arthritis and periodontitis, exhibited higher FAPI uptake than those in purely degenerative diseases. The authors established a cumulative cutoff SUV_max_ of 4.7 with a sensitivity and specificity of 74.3% and 68.3%, respectively, to distinguish between malignant and benign lesions. Nevertheless, that cohort showed a large overlap in SUV_max_ values as well. Dabir et al. (30) conducted a study with a cohort of 155 patients with cancer, evaluating benign lesions and inflammatory processes in various sites. The mean SUV_max_ of benign lesions tended to be lower than that of malignant lesions (4.2 vs. 10.6, respectively; *P* < 0.001). The overall SUV_max_ cutoff value of 5.5 displayed sensitivity, specificity, accuracy, and an area under the curve of 78.8%, 85.1%, 82.0%, and 0.89%, respectively. The authors, however, pointed out that qualitative parameters, such as other imaging findings, special location, and clinical data, must be considered. Further, these data must be contextualized within the lack of histologic proof in many cases.

Current evidence highlights the approach of qualitative lesion discrimination rather than focusing on the use of semiquantitative parameters in FAP-targeted imaging. Given the variety of FAP ligand–uptake patterns across malignancies, no general cutoff values in terms of SUV metrics appear to be feasible.

### Next Step for Standardizing the Interpretation and Reporting of Findings from FAP-Targeted Imaging

The standardized framework of the American College of Radiology RADS seems to provide a solid backbone for the development of a reporting schema for FAP images for use in regular clinical care and research. The essential characteristics of RADS are its prerequisites, such as minimum disease-focused technical specifications, a user-friendly lexicon with a coded ordinal scale for expressing the probability of disease or disease aggressiveness, guidance for organizing the radiology report, and recommendations to clinicians (31). In light of the unmet needs regarding FAP PET image interpretation, as well as the advantages of a RADS framework, we propose a novel structured reporting system based on the RADS framework, termed *FAP-RADS version 1.0*, for reporting findings from FAP-targeted imaging.

Our proposal is designed to be modality-nonspecific, as it may be applied for both FAP SPECT and FAP PET radiotracers. The proposed framework integrates not only molecular imaging parameters but also morphologic criteria and medical history, underscoring its applicability across multiple modalities, including PET/CT or PET/MRI. Moreover, in line with the principles of RADS frameworks, our proposal foresees the clear categorization of suspected target lesions separately and mentioning an overall impression of the imaging study by a FAP-RADS score across various clinical scenarios. Beyond that, this approach reinforces molecular imaging diagnostics by aligning with a composite reference standard from the available extensive literature, which allows strong wording in imaging reports in regular clinical care without necessarily obtaining histopathologic confirmation.

### Overview of FAP-RADS Version 1.0

Table 1 provides an overview of FAP-RADS version 1.0. The following sections provide guidance for each specific category of the reporting system, accompanied by a figure presentation.

**TABLE 1.** Overview of FAP-RADS Version 1.0 for FAP-Targeted Imaging

Score	Description
FAP-RADS 1 (benign)	Typical benign FAP ligand uptake in normal organs (such as joints or background activity)
FAP-RADS 2 (likely benign)	Equivocal lesions with slight-to-intense FAP ligand uptake* with no suspected anatomic correlate (degenerative or inflammatory changes or posttraumatic or postinterventional changes)
FAP-RADS 3 (suggestive of malignancy)	
FAP-RADS 3A	Equivocal uptake in sites typical for the “suspected malignancy” without anatomic correlate; follow-up imaging recommended
FAP-RADS 3B	Equivocal FAP ligand uptake in sites typical for the suspected malignancy with (un-)specific anatomic correlate **or** highly suspected lesions (morphologic correlate) with no significant FAPI uptake; follow-up imaging or biopsy recommended
FAP-RADS 4 (malignancy highly likely)	Intense FAP ligand uptake* in sites typical for suspected malignancy with unspecific or no anatomic correlate; biopsy recommended
FAP-RADS 5 (consistent with malignancy)	Intense FAP ligand uptake* in sites typical for the suspected malignancy with corresponding findings on conventional imaging; biopsy or initiation of therapy (surgery, radiotherapy, or chemotherapy) recommended
Overall RADS score	Defined by the highest FAP-RADS score of any of the individual target lesions

* Intense FAP ligand uptake defined as 3-fold of SUV_max_ in blood pool in descending aorta.

#### FAP-RADS 1

**FIGURE 1.**
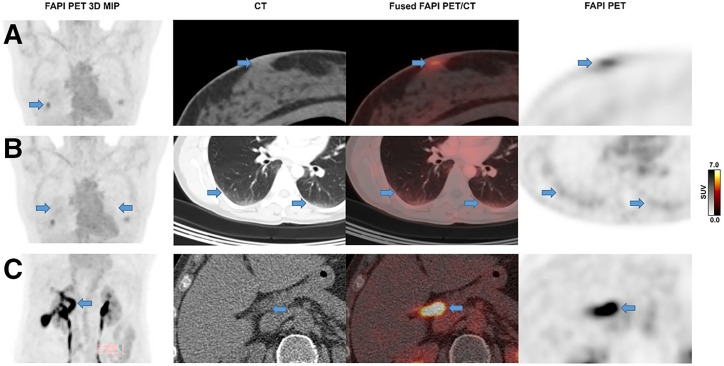
Examples of FAP-RADS 1 lesions. Upper row (A) shows FAPI uptake of mammillae in 37-y-old woman. Symmetric distribution of focal FAPI uptake and absence of suspicious morphologic findings allow stratification as benign lesion. Middle row (B) depicts nonspecific FAPI uptake of dependent densities in dorsal lower lungs of same patient due to gravity-dependent, relative blood pooling. Lower row (C) shows highly intense FAPI uptake in biliary tract attributed to biliary excretion of FAP tracer in 72-y-old woman. Intensity may vary among FAPI compounds. MIP = maximum-intensity projection.

FAP-RADS 1 lesions are typically benign lesions, based on their well-known anatomic, functional, or histologic features. Any slightly increased FAPI uptake of such lesions is not to be considered pathologic. Examples include diffusely or focally increased uptake in normal organs and tissues (e.g., biliary tract, nipples, joints) and increased FAPI uptake in dorsal segments of lower lung lobes, which otherwise would mimic malignancy (Fig. 1). The aim of this category is to provide a basis for both the referring physician and imaging expert, who may need a conclusive statement regarding a FAPI-avid lesion that is a well-known benign phenomenon. All typical benign lesions should be classified as FAP-RADS 1, regardless of the degree of FAP ligand uptake.

#### FAP-RADS 2

**FIGURE 2.**
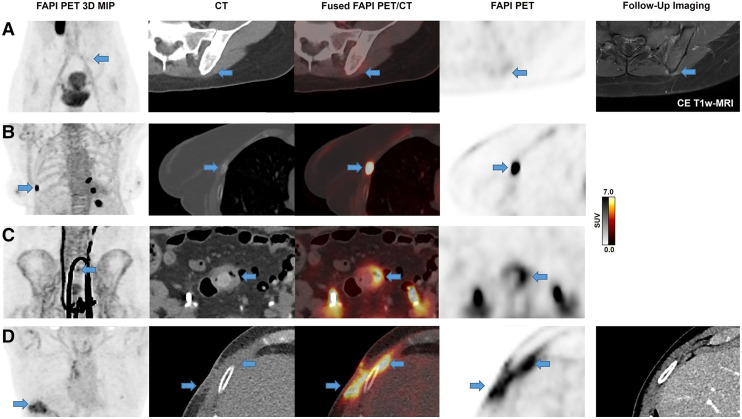
Examples of FAP-RADS 2 lesions. Images in upper row (A) show discrete FAPI uptake along left posterior ileum with corresponding contrast enhancement on T1-weighted (T1w) MRI, consistent with inflammation or enthesitis. Second row (B) illustrates multifocal intense FAPI uptake on both sides of rib cage in posttraumatic setting with correlating rib fractures on morphologic imaging. Third row (C) shows example of typical uncomplicated sigmoid diverticulitis with intensive FAPI uptake. Clinical background information and CT imaging should be examined carefully to avoid missing neoplasia, which would lead to upgraded FAP-RADS category. Bottom row (D) illustrates typical FAPI uptake pattern after invasive medical intervention on right chest wall, which can only be recognized with medical history but was confirmed on follow-up CT imaging. MIP = maximum-intensity projection.

FAP-RADS 2 lesions are highly likely benign, regardless of the FAP ligand–uptake intensity, based on anatomic appearance and medical history, with no conclusive negative histologic evidence or completely pathognomonic appearance on CT. Another identifying trait is that the suspected lesion or FAP ligand uptake is atypical for the malignancy of focus. Some examples include joint arthritis or focal FAPI-avid tissue changes after a medical intervention or implantation of a medical FB. Even though the differences between FAP-RADS 1 and FAP-RADS 2 may seem somewhat ambiguous, depending on the experience or interpretation of the reader, FAP-RADS 2 lesions are characterized by the presence of intensified FAP ligand uptake. Figure 2 illustrates some typical examples in this regard, including degenerative and inflammatory changes, posttraumatic changes, and postinterventional changes with no malignancy-suspected anatomic correlate.

#### FAP-RADS 3A and 3B

**FIGURE 3.**
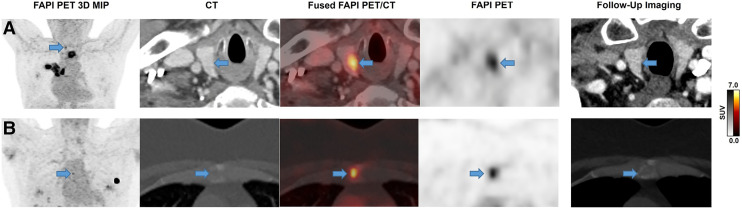
Row A depicts FAP-RADS 3A lesion in 73-y-old patient with non–small cell lung cancer with moderate focal FAPI uptake in right lobe of thyroid. There was no morphologic correlate on conventional follow-up imaging 6 mo later, leading to retrospective classification as unspecific FAPI uptake. Row B shows focal moderate FAPI uptake in manubriosternal joint with accompanying nonspecific CT correlate, meeting FAP-RADS 3B criteria, without any change on follow-up imaging 9 mo later. MIP = maximum-intensity projection.

FAP-RADS 3 lesions are suggestive of malignancy and require further clarification via follow-up imaging or biopsy (Fig. 3). A typical FAP-RADS 3 lesion would mostly exhibit faint-to-moderate FAPI uptake that may be accompanied by a nonspecific morphologic correlate for the malignancy of interest. For lesions suggestive of malignancy, we defined 2 subcategories within FAP-RADS 3: FAP-RADS 3A and FAP-RADS 3B.

A typical FAP-RADS 3A lesion exhibits focal faint-to-moderate FAPI uptake without any anatomic correlate. Considering the tenets of molecular imaging, we anticipate a substantial number of lesions of interest in FAP-targeted imaging fitting into this subcategory.

FAP-RADS 3B lesions, however, have a focal faint-to-moderate FAPI uptake with an accompanied (non-)specific morphologic correlate for the malignancy of interest. This subcategory includes lesions with highly suggestive or typical morphologic correlates for malignancy without FAPI uptake. Given the increased likelihood of malignancy, FAP-RADS 3B suggests the possibility of biopsy in addition to serial follow-up imaging. In regular clinical care, a biopsy may be preferred over follow-up imaging if it would have a significant effect on subsequent patient management. If the results of the subsequent serial follow-up imaging increase the likelihood of malignancy for the particular lesion, then the FAP-RADS category should be upgraded to FAP-RADS 4 or FAP-RADS 5 (32). Accordingly, if the subsequent imaging follow-up rules out the malignancy, then the lesion of interest should be downgraded to FAP-RADS 1 or FAP-RADS 2. The clinical context should have priority in the interpretation of lesions. For example, a lesion formally fitting the criteria for FAP-RADS 3 in a patient with extensive metastatic disease should be upgraded to FAP-RADS 4 or FAP-RADS 5, even if the prerequisites for this categorization are not fully met (33).

To date, no FAPI radiotracer has been approved by the U.S. Food and Drug Administration or European Medicines Agency; thus, the use of FAPI imaging has been restricted to clinical trials or individual cases for compassionate use. Therefore, we refrain from giving strong recommendations regarding the frequency or timing of follow-up FAPI scans at this time. As the data continue to mature and more robust clinical trials are published, we hope to refine the follow-up recommendations to appropriately match the emerging data.

#### FAP-RADS 4

**FIGURE 4.**
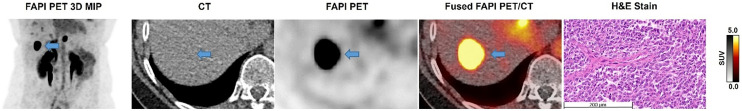
Example of FAP-RADS 4 lesion, depicting focal intense FAPI uptake in right lobe of liver without specific morphologic correlate. Biopsy-confirmed solitary fibrous tumor metastasis. H&E = hematoxylin and eosin; MIP = maximum-intensity projection.

FAP-RADS-4 lesions typically exhibit a high or intense FAPI uptake with a nonspecific or unclear morphologic correlate in a typical site for the malignancy of interest. The subsequent logical step in clinical management could be biopsy for a timely histologic validation (Fig. 4), if such validation would alter clinical management. In many instances, the presence of a FAP-RADS 4 lesion will be de facto evidence of a site of cancer, and histologic validation may not be necessary.

#### FAP-RADS 5

**FIGURE 5.**
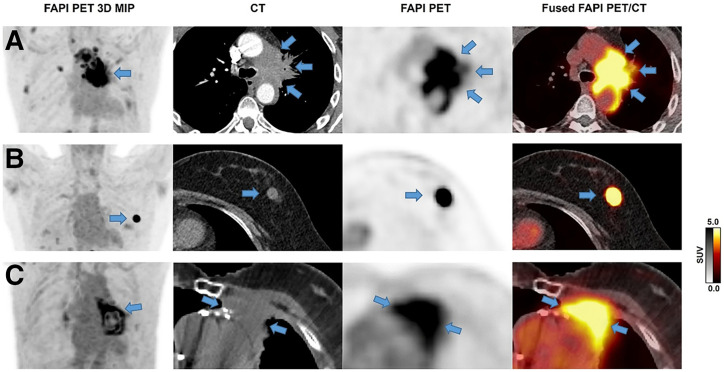
Examples of FAP-RADS 5 lesions. Rows A and C depict biopsy-confirmed cases of central non–small cell lung cancer infiltrating mediastinum. Row B shows metastatic nodule of solitary fibrous tumor in left breast, which was also confirmed histologically. All malignant lesions are characterized by intense FAPI uptake with typical morphologic correlates. MIP = maximum-intensity projection.

FAP-RADS 5 underscores consistency with malignancy for the lesion of interest, as such lesions exhibit classic findings of both significant FAPI uptake and a clear morphologic correlate at a typical site for the malignancy of interest (Fig. 5). Given the utmost probability of malignancy for this category of lesions, the subsequent step in clinical management may involve histologic validation, if clinically warranted, or initiation of therapy (e.g., surgery, chemoradiotherapy), if appropriate.

#### Overall FAP-RADS Score

As previously discussed, we strongly recommend that imaging experts provide an overall impression by reporting the highest FAP-RADS score of any individual lesion or any FAP-RADS score that would require attention from the treating clinician to select further work-up. The final report should not include the FAP-RADS categorization of more than 5 key lesions. A 3-fold SUV_max_ of the blood pool in the descending aorta is proposed as the visual benchmark for distinguishing intense from equivocal FAP ligand uptake of the lesion of interest. This concept would ensure greater clinical impact of FAP-targeted imaging among referring physicians, as it would provide better comprehensibility of reports and enhance the clinical decision-making process both for clinicians and imaging experts.

## DISCUSSION

Considering the rapid growth of evidence supporting FAP-targeted imaging, the research and clinical landscape mandate a standardized, reliable reporting system. This would not only enhance communication among physicians and strengthen the robustness of FAP-targeted imaging but also increase the clinical impact of FAP-targeted imaging by ensuring a standardized interpretation of findings of interest during longitudinal follow-up. This reporting system could also pave the way for the conduct of multicenter trials with large patient cohorts involving FAP-targeted imaging, as the efficacy and employment of FAPI imaging in epithelial malignancies are still complex and diverse topics. Our comprehensive literature search, combined with our grounded clinical expertise, has led us to the proposal of the FAP-RADS version 1.0, which aims to address those unmet needs. FAP-RADS version 1.0 shares the fundamental elements of the molecular imaging RADS framework, as that framework has aimed to be integrable with the overall goals of RADS: user-friendliness, easy application to regular clinical care, and ready comprehensibility for nonimaging experts (31,34).

One of the main goals during the design of the FAP-RADS version 1.0 was to ensure its broad applicability across numerous tumor entities with varying FAP ligand–uptake patterns. Moreover, this new scoring system was designed to be nonspecific to imaging modality and radiotracer compound structure, making it likely suitable for the interpretation of both FAP PET and FAP SPECT radiotracers.

This study had limitations. It lacked data from large, prospective, multicenter trials; however, the conduct of such large, multicenter studies necessitates reliable reporting systems. The rollout of this reporting system may help to overcome that obstacle. For this study, we used the data from studies predominantly deploying FAP PET tracers, since the current evidence is dominated by data involving such agents. Nevertheless, we assume that the concept of this scoring system is also applicable to FAP SPECT ligands. Future data obtained after the roll-out of this scoring system will allow us to assess its clinical impact and prognostic value, which could lead to adjustments to the existing framework. That approach is typical in the development process of classification systems for imaging. During the process of evidence synthesis, we assumed the interchangeability of various FAP-targeted ligands on the basis of our experience and data from the literature. The qualitative nature of interpretations with this scoring system does not mandate a strong consideration of SUV metrics, potentially enhancing its applicability across numerous FAP ligands with varying pharmacokinetics. In this regard, this approach mandates prioritization of the clinical context for accurate interpretation of the lesions. The 5-point scale of FAP-RADS version 1.0 gives an increasing probability of malignancy with higher numbers.

The intrinsic nuance of any molecular imaging RADS has proven difficult to approach from a machine-learning or artificial intelligence perspective. FAP-RADS presents unique challenges, given the wide array of oncologic and nononcologic entities that exhibit FAP ligand uptake and the potential for the imaging agents to identify multiple overlapping malignancies. The field of nuclear medicine would do well to focus on the development of large, annotated datasets that can underlie a robust artificial intelligence approach to automating FAP-RADS as well as providing an excellent resource for training FAPI-naïve readers (35,36).

## CONCLUSION

The proposed FAP-RADS version 1.0 is a comprehensive and flexible framework that has the potential to enhance the quality and consistency of FAP-targeted imaging interpretation in clinical practice. Its application could lead to better research outcomes, broader acceptance within the oncologic community, and streamlined regulatory processes related to FAP-based radiotracer approval.

## DISCLOSURE

Frederik Giesel has a patent application for quinolone-based FAP-targeting agents for imaging and therapy in nuclear medicine, shares a consultancy group for iTheranostics, and is an advisor at ABX, Telix, α-Fusion, and SOFIE Biosciences. Steven Rowe is a coinventor on a patent for quinolone-based FAP-targeting agents and consultant for Lantheus, Telix, and Blue Earth Diagnostics. Rudolf Werner received speaker honoraria from Novartis/AAA and PentixaPharm and reports advisory board work for Novartis/AAA and Bayer. Adrien Holzgreve reports compensation for scientific consulting by ABX and is funded by the Deutsche Forschungsgemeinschaft (DFG, German Research Foundation; 545058105). Ken Herrmann received personal fees from Bayer, SIRTEX, Adacap, Curium, Endocyte, IPSEN, Siemens Healthineers, GE HealthCare, Amgen, Novartis, ymabs, Aktis, Oncology, and Pharma15, as well as personal and other fees from SOFIE Biosciences, nonfinancial support from ABX, and grants and personal fees from BTG, all of which are outside the submitted work. No other potential conflict of interest relevant to this article was reported.

## Notes

KEY POINTS **QUESTION:** Is there a systematic evaluation strategy for FAP-targeted imaging? **PERTINENT FINDINGS:** The critical evaluation of existing data on FAP PET and our expertise allowed the introduction of a scientifically substantiated, unified language in a new standardized reporting system, FAP-RADS version 1.0. **IMPLICATIONS FOR PATIENT CARE:** This standardized language underscores the comprehensibility of imaging reports in the medical community and can enhance its acceptance as well. Moreover, this will provide a solid framework for the design and interpretation of multicenter prospective trials, which may facilitate the approval and reimbursement of FAP ligands by regulatory bodies.
